# Blindness Survey Methods: Response from Sudan Study Authors

**DOI:** 10.1371/journal.pmed.0040086

**Published:** 2007-02-27

**Authors:** Jeremiah Ngondi, Francis Ole-Sempele, Alice Onsarigo, Ibrahim Matende, Samson Baba, Mark Reacher, Fiona Matthews, Carol Brayne, Paul M Emerson

We thank the authors [1,2] for the two perspectives on our articles [3,4]. Our study estimated the prevalence of blindness in Mankien at 4%, which Kuper and Gilbert describe as being “beyond the range” of the studies reviewed by Pascolini et al. [5]. The review did not include any studies from the ten states that compose southern Sudan. The nearest surveys reported were conducted in 1998 in Al-Ginena province of Southern Darfur—which is within the 16 northern states of Sudan governed from Khartoum, and was not directly affected by the war in the south. The Al-Ginena studies show a blindness prevalence of 3.2% in all ages [6,7]. Yet despite the geographical proximity, two decades of civil war in the south were accompanied by the absence of a health infrastructure, and no preventive health services to speak of, which makes southern Sudan unique. Comparisons with other parts of Sudan or with other countries are probably not justified or meaningful.

Our survey was conducted in Mankien, which was anecdotally known to be endemic for severe blinding trachoma, and this was subsequently confirmed by our trachoma survey, which showed an overall prevalence of trichiasis and bilateral corneal opacity of 9.6% and 3.1%, respectively [3]. The prevalence of blinding cataract in Mankien was consistent with expectation, and would presumably have been higher had there been a systematic over-sampling of the blind. It is the prevalence of blinding trachoma that sets the population apart from all others reported and reviewed by Pascolini et al. These survey data from Mankien are extremely valuable in that they demonstrate the way uncontrolled trachoma can ravage inaccessible and underserved communities who have, quite literally, been off the map until recently. The war affected the whole of southern Sudan, and extremely high levels of active trachoma and trichiasis have been observed in all the areas that we have managed to survey in recent years [8]. Although not generalizable to the ten southern states, these data from Mankien are probably indicative of the overall situation in southern Sudan.

We acknowledge the survey limitations highlighted by Kuper and Gilbert, and have addressed them in the discussion. Our sampling methodology has been used in similar surveys in Kenya [9], Bangladesh [10], Tehran [11], Cameroon [12], and Pakistan [13]. Use of basic eye examination technique by auxiliary health-care workers in blindness surveys has been suggested for settings without ophthalmologists [14], which is entirely consistent with what the integrated eye care workers are trained to do. Kuper and Gilbert's difficulty appears to stem from the findings, rather than the methodology. They offer two interpretations to explain the data, whilst not acknowledging the most parsimonious explanation, and that is that these data are an accurate reflection of the situation on the ground; we cannot risk not accepting this. The international community must rise to the challenge of planning and offering service for blindness prevention interventions in these desperately needy communities. We should not let an academic argument as to whether the prevalence of blindness is really 4% or whether it is, perhaps, a little lower be the basis for continued neglect.

We fully agree with both Kuper and Gilbert, and Buchan that there is need for a concerted effort to survey the entire region, provide resources, and deliver services to the marginalized and poverty stricken communities in southern Sudan.
